# A new fossil piddock (Bivalvia: Pholadidae) may indicate estuarine to freshwater environments near Cretaceous amber-producing forests in Myanmar

**DOI:** 10.1038/s41598-021-86241-y

**Published:** 2021-03-23

**Authors:** Ivan N. Bolotov, Olga V. Aksenova, Ilya V. Vikhrev, Ekaterina S. Konopleva, Yulia E. Chapurina, Alexander V. Kondakov

**Affiliations:** 1 Northern Arctic Federal University, Northern Dvina Emb. 17, 163002 Arkhangelsk, Russia; 2 N. Laverov Federal Center for Integrated Arctic Research, Ural Branch, Russian Academy of Sciences, Northern Dvina Emb. 23, 163000 Arkhangelsk, Russia

**Keywords:** Taxonomy, Zoology, Biogeography, Palaeoecology

## Abstract

The lower Cenomanian Kachin amber from Myanmar contains a species-rich assemblage with numerous plant and animal fossils. Terrestrial and, to a lesser degree, freshwater species predominate in this assemblage, while a few taxa with marine affinities were also discovered, e.g. isopods, ammonites, and piddocks. Here, we describe the Kachin amber piddock †*Palaeolignopholas kachinensis* gen. & sp. nov. It appears to be an ancestral stem lineage of the recent *Lignopholas* piddocks, which are estuarine to freshwater bivalves, boring into wood and mudstone rocks. Frequent occurrences and high abundance of †*Palaeolignopholas* borings and preserved shells in the Kachin amber could indicate that the resin-producing forest was partly situated near a downstream (estuarine to freshwater) section of a river. Multiple records of freshwater invertebrates (caddisflies, mayflies, stoneflies, odonates, and chironomids) in this amber could also manifest in favor of our paleo-environmental reconstruction, although a variety of local freshwater environments is known to occur in coastal settings.

## Introduction

Piddocks (Bivalvia: Pholadidae) are a primarily marine family of bivalves^1–4^. These clams represent a group of ecosystem engineers driving macrobioerosion and sedimentation processes in marine environments, and increasing surface complexity of rocks, coral reefs, wood, and other hard substrates^5–8^. Several piddocks such as members of the recent genera *Lignopholas* Turner, 1955 and *Martesia* Sowerby 1824 can tolerate a wide range of salinity, being common inhabitants of estuarine environments^9–12^. Moreover, at least two primarily estuarine *Lignopholas* species can establish permanent populations in freshwater habitats, i.e. the wood-borer *L. rivicola* (Sowerby, 1849)^11^, and the rock-borer *L. fluminalis* (Blanford, 1867)^13,14^.

Club-shaped (clavate) borings produced by piddocks are common trace fossils occurring in a broad variety of substrates^5^, including amber from Columbia, Mexico, Myanmar, Lebanon, France, and Spain^15^. Bivalve shells and borings in Cretaceous amber from northern Myanmar were initially identified as plant antheridia or fungal sporangia, and the first careful reconstruction of this fossil, showing immature shell with interior and exterior futures was presented^16^. Later, the piddock trace fossils were described as sporocarps of the non-gilled hymenomycete *Palaeoclavaria burmitis* Poinar & Brown, 2003^17,18^. Further researchers recorded fossil remains of piddock shells inside the borings and those “floating” in the amber^15,19,20^. Based on conchological characters, these shells were considered belonging to the subfamily Martesiinae^19^, and, more specifically, to the genus *Martesia*^15^.

This study aims to (1) describe a new genus and species of Mesozoic piddocks; (2) assess the taxonomic placement of this taxon and its relationships with other genera of the Martesiinae; and (3) discuss taphonomic issues regarding aquatic environments near the early Cenomanian amber-producing forest in northern Myanmar.

## Results

**Figure 1:**
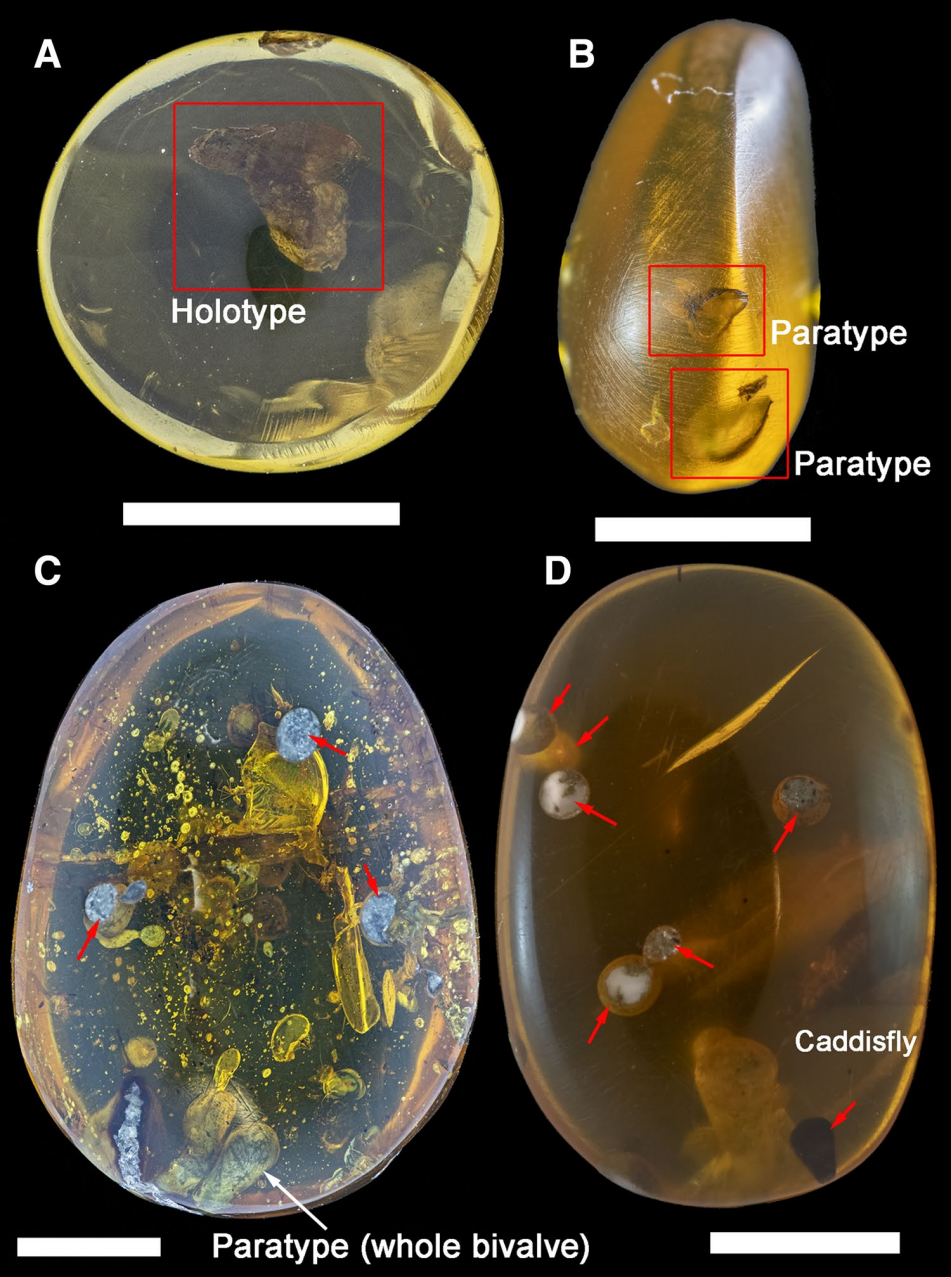
Lower Cenomanian Kachin amber samples with specimens and borings of †*Palaeolignopholas kachinensis* gen. & sp. nov. from northern Myanmar used in this study. (**A**) RMBH biv1115 (frontal view with the holotype). (**B**) RMBH biv1101 (lateral view with two paratypes and a shell fragment). (**C**) RMBH biv1116 (frontal view with the fossilized paratype). (**D**) RMBH biv1100 (frontal view with borings). The red frames indicate position of the type specimens (holotype and some paratypes). The red arrows indicate bivalve borings. Scale bars = 5 mm. (Photos: Ilya V. Vikhrev).

**Figure 2:**
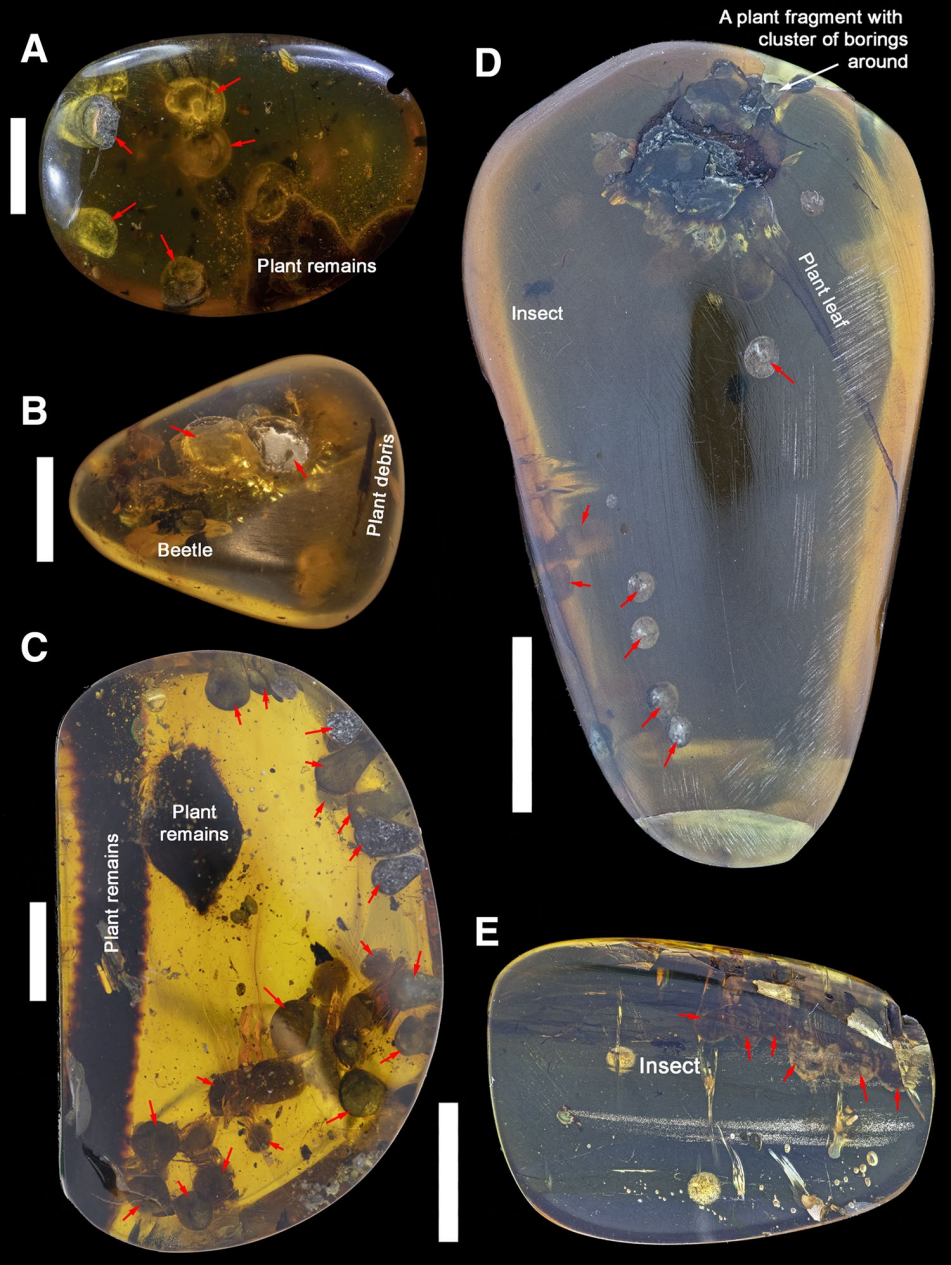
Lower Cenomanian Kachin amber samples with borings of †*Palaeolignopholas kachinensis* gen. & sp. nov. from northern Myanmar used in this study. (**A**) RMBH biv1102 (frontal view). (**B**) RMBH biv1103 (frontal view). (**C**) RMBH biv1114 (frontal view). (**D**) RMBH biv1118 (frontal view). (**E**) RMBH biv1117 (frontal view). The red arrows indicate bivalve borings. Scale bars = 5 mm. (Photos: Ilya V. Vikhrev).

Altogether nine polished pieces of the lower Cenomanian Kachin amber from northern Myanmar (Figs. 1A–D, 2A–E) were examined in this study (depository: Russian Museum of Biodiversity Hotspots, N. Laverov Federal Center for Integrated Arctic Research of the Ural Branch of the Russian Academy of Sciences, Arkhangelsk, Russia). A brief description of each amber piece is given below.

RMBH biv1115: Size 8.5 × 5.8 × 8.1 mm (Fig. 1A). Inclusions: articulated shell of †*Palaeolignopholas kachinensis* gen. & sp. nov., “floating” in the resin (the holotype).

RMBH biv1101: Size 15.6 × 6.4 × 11.5 mm (Fig. 1B). Inclusions: two complete articulated shells (paratypes) and a shell fragment of †*Palaeolignopholas kachinensis* gen. & sp. nov., “floating” in the resin.

RMBH biv1116: Size 22.5 × 8.3 × 16.5 mm (Fig. 1C). Inclusions: fossilized shell of †*Palaeolignopholas kachinensis* gen. & sp. nov. (paratype), borings of this species (filled with fine gray sand), unidentified fly specimens (Insecta: Diptera), and unidentified organic fragments (probably, plant debris).

RMBH biv1100: Size 17.5 × 4.9 × 12.0 mm (Fig. 1D). Inclusions: borings of †*Palaeolignopholas kachinensis* gen. & sp. nov. (filled with fine gray sand), and an unidentified caddisfly specimen (Insecta: Trichoptera).

RMBH biv1102: Size 15.6 × 5.1 × 12.7 mm (Fig. 2A). Inclusions: borings of †*Palaeolignopholas kachinensis* gen. & sp. nov. (filled with fine gray sand), and unidentified organic fragments (probably, plant debris).

RMBH biv1103: Size 19.6 × 4.7 × 14.3 mm (Fig. 2B). Inclusions: borings of †*Palaeolignopholas kachinensis* gen. & sp. nov. (filled with fine gray sand), an unidentified beetle specimen (Insecta: Coleoptera), and unidentified organic fragments (probably, plant debris).

RMBH biv1114: Size 33.1 × 7.8 × 21.7 mm (Fig. 2C). Inclusions: multiple borings of †*Palaeolignopholas kachinensis* gen. & sp. nov. (filled with fine gray sand), and unidentified plant remains.

RMBH biv1118: Size 25.1 × 8.4 × 14.3 mm (Fig. 2D). Inclusions: separate borings of †*Palaeolignopholas kachinensis* gen. & sp. nov. (filled with fine gray sand), a plant fragment with a cluster of borings around, and an unidentified insect specimen.

RMBH biv1117: Size 15.5 × 3.9 × 10.7 mm (Fig. 2E). Inclusions: borings of †*Palaeolignopholas kachinensis* gen. & sp. nov. (filled with fine gray sand), and an unidentified insect specimen.

Additionally, six amber samples containing adult and sub-adult specimens of †*Palaeolignopholas kachinensis* gen. & sp. nov. were examined using photographs in published works as follows: BMNH 20205 (Department of Palaeontology, Natural History Museum, London, UK)^15^, NIGP 169623 and NIGP 169624 (Nanjing Institute of Geology and Palaeontology, Chinese Academy of Sciences, Nanjing, China)^20^, RS.P1450 (Ru D. A. Smith collection, Kuala Lumpur, Malaysia)^19^, and AMNH (Division of Invertebrates, American Museum of Natural History, New York, NY, United States of America)^16^.

Based on morphological analyses of the fossil piddock shells, it was found to be a genus and species new to science, which is described here.

### Systematic paleontology

Phylum Mollusca Linnaeus, 1758

Class Bivalvia Linnaeus, 1758

Family Pholadidae Lamarck, 1809

Subfamily Martesiinae Grant & Gale, 1931

### †*Palaeolignopholas* gen. nov

LSID: http://zoobank.org/urn:lsid:zoobank.org:act:1D686DCE-A5E9-41DA-9504-2EC58C93D988

Type species: †*Palaeolignopholas kachinensis* gen. & sp. nov.

Etymology. This name is derived from the prefix ‘Palaeo-’ (ancient), and ‘-lignopholas’, the name of a recent genus of estuarine and freshwater piddocks boring into wood, mudstone rocks, brickwork, laterites, etc.^11,13^. Masculine in gender.

Diagnosis. The new monotypic genus is conchologically similar to several other piddock genera such as *Lignopholas*, *Martesia*, and *Diplothyra* Tryon 1862 but can be distinguished from these taxa by the following combination of characters: mesoplax relatively small, triangular, divided longitudinally, posterior slope without concentric sculpture, sculptured valve with concave parallel ridges (*Martesia*-like “rasping teeth”) curved anteriorly, periostracal lamellae dense, fine, hair-like. The fossil genus †*Opertochasma* Stephenson, 1952 shares a divided mesoplax but it clearly differs from both †*Palaeolignopholas* gen. nov. and *Lignopholas* by having two radial grooves on the shell surface^21^.

Distribution. Kachin State, northern Myanmar; Upper Cretaceous (lower Cenomanian)^15,19,22^.

Comments. Both †*Palaeolignopholas* gen. nov. and *Lignopholas* appear to be closely related to each other because they share a longitudinally divided mesoplax and periostracal lamellae, which are considered diagnostic features distinguishing this clade from *Martesia* + *Diplothyra*. Based on available conchological characters, we assume that †*Palaeolignopholas* gen. nov. might be placed on the ancestral stem lineage of the *Lignopholas* clade, although a possibility of homeomorphy could not entirely be excluded.

### †*Palaeolignopholas kachinensis* gen. & sp. nov

**Figure 3:**
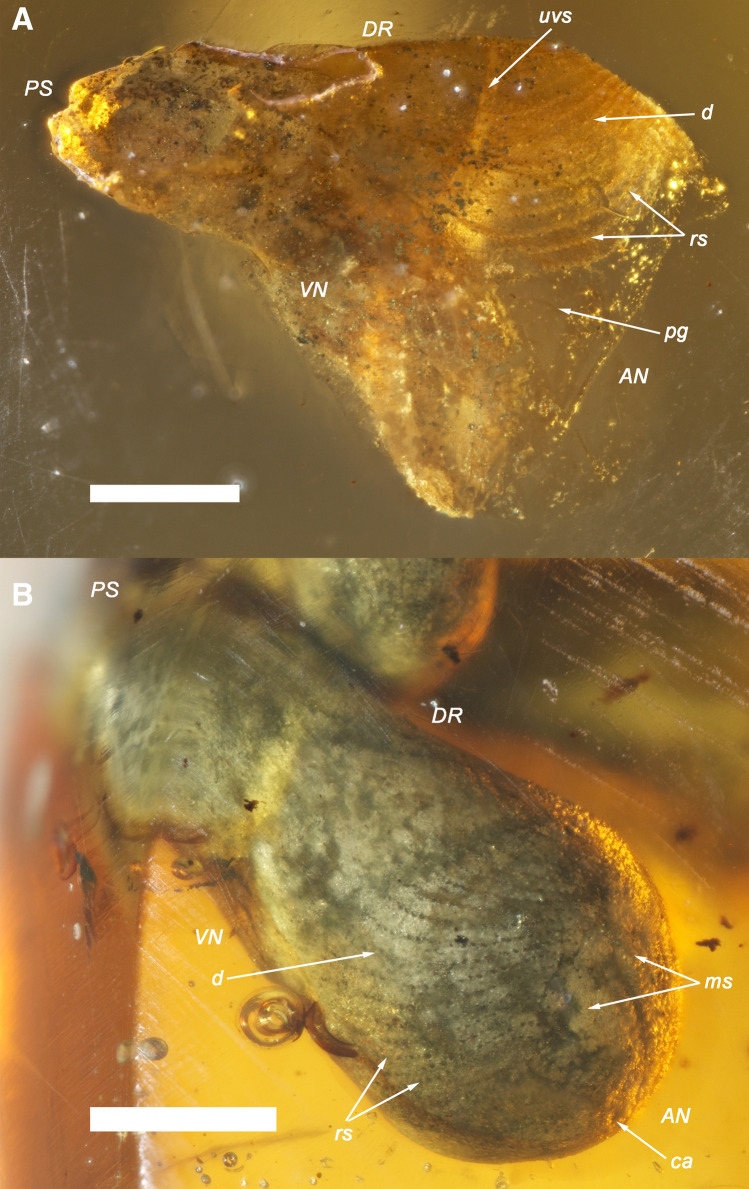
Holotype and a paratype of †*Palaeolignopholas kachinensis* gen. & sp. nov. from lower Cenomanian Kachin amber, northern Myanmar. (**A**) Holotype: ventro-lateral view of articulated shell. (**B**) Paratype: anterio-lateral view of fossilized shell. *VN* ventral margin; *DR* dorsal margin; *AN* anterior margin; *PS* posterior margin; *d* disc; *rs* rasping surface of the valve; *uvs* umbonal ventral sulcus; *pg* pedal gape; *pl* periostracal lamellae. Scale bars = 500 µm. (Photos: Ilya V. Vikhrev).

 = Plant Antheridia or Fungal Sporangia indet. sensu Grimaldi et al. (2002): 9, fig. 2a,b (bivalve specimens), fig. 3 (borings), fig. 5 (shell reconstruction of an immature specimen), figs. 6 and 7 (SEMs of borings surface showing rasped ornament at different magnifications)^16^.

 = *Palaeoclavaria burmitis* Poinar & Brown (2003): 765, figs. 1–4 (borings) [this fungal taxon was introduced using a trace fossil (boring) as the holotype]^17^; Poinar (2016): 2, figs. 10, 15, 16 (borings)^18^.

 = Martesiinae indet. sensu Smith & Ross (2018): 4, figs. 1a–c, 2a,b, 3a–d (borings), 4a,b, 5a–e (bivalve specimens)^19^.

 = Pholadidae indet. sensu Mao et al. (2018): 99, figs. 8a–f (borings), 8g,h (bivalve specimens)^20^.

 = *Martesia* sp. 2 sensu Mayoral et al. (2020): 10, figs. 4a (borings), 7b, 8a–l (bivalve specimens)^15^.

 = Pholadidae indet. sensu Balashov (2020): 6^23^.

**Figure 4:**
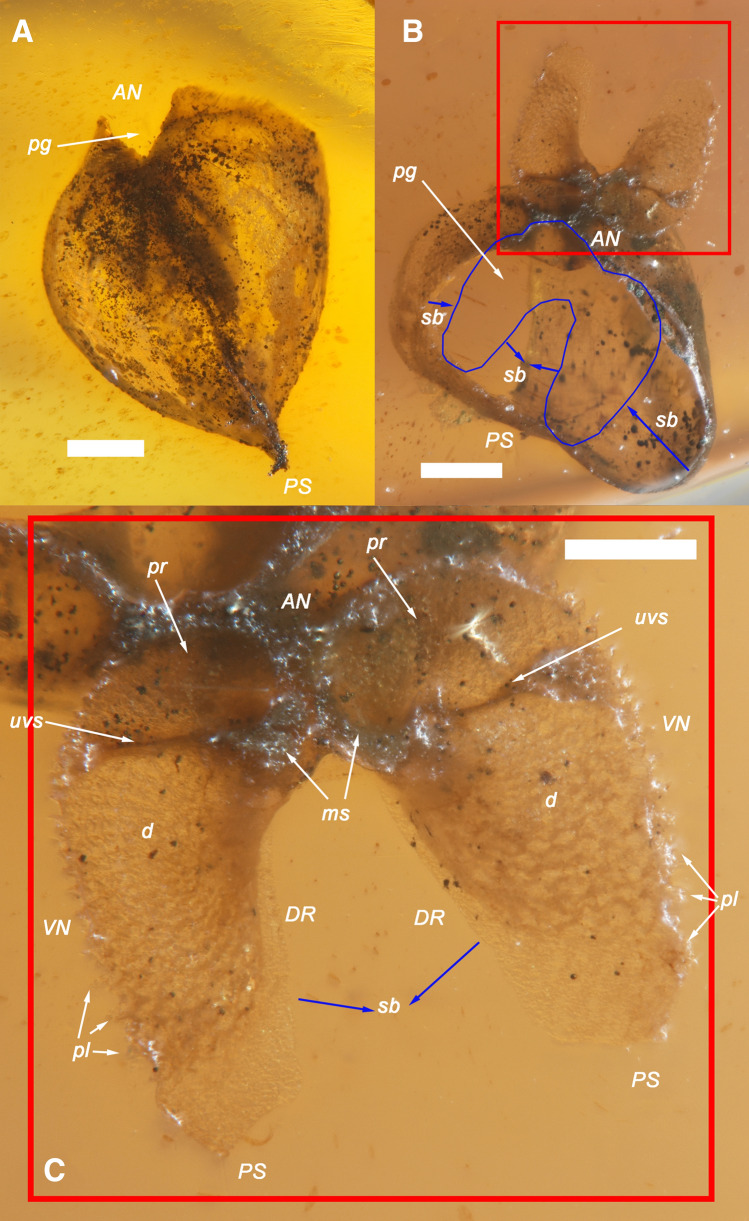
Paratypes of †*Palaeolignopholas kachinensis* gen. & sp. nov. from lower Cenomanian Kachin amber, northern Myanmar. (**A**) Paratype: dorsal view of articulated shell. Scale bar = 500 µm. (**B**) Paratype: dorsal view of articulated shell. The detached and deflected umbonal paired fragment of the valves is framed by red square. The blue contour indicates the lifetime position of this fragment. The blue arrows show the shell breakages. Scale bar = 200 µm. (**C**) Umbonal paired fragment of the holotype valves (inner view). The blue arrows show the shell breakage. Scale bar = 200 µm.  *VN* ventral margin; *DR* dorsal margin; *AN* anterior margin; *PS* posterior margin; *ms* longitudinally divided mesoplax (inner view); *pr* prora; *d* disc; *rs* rasping surface of the valve; *uvs* umbonal ventral sulcus; *pg* pedal gape; *pl* periostracal lamellae; *sb* shell breakage. (Photos: Ilya V. Vikhrev).

**Figure 5:**
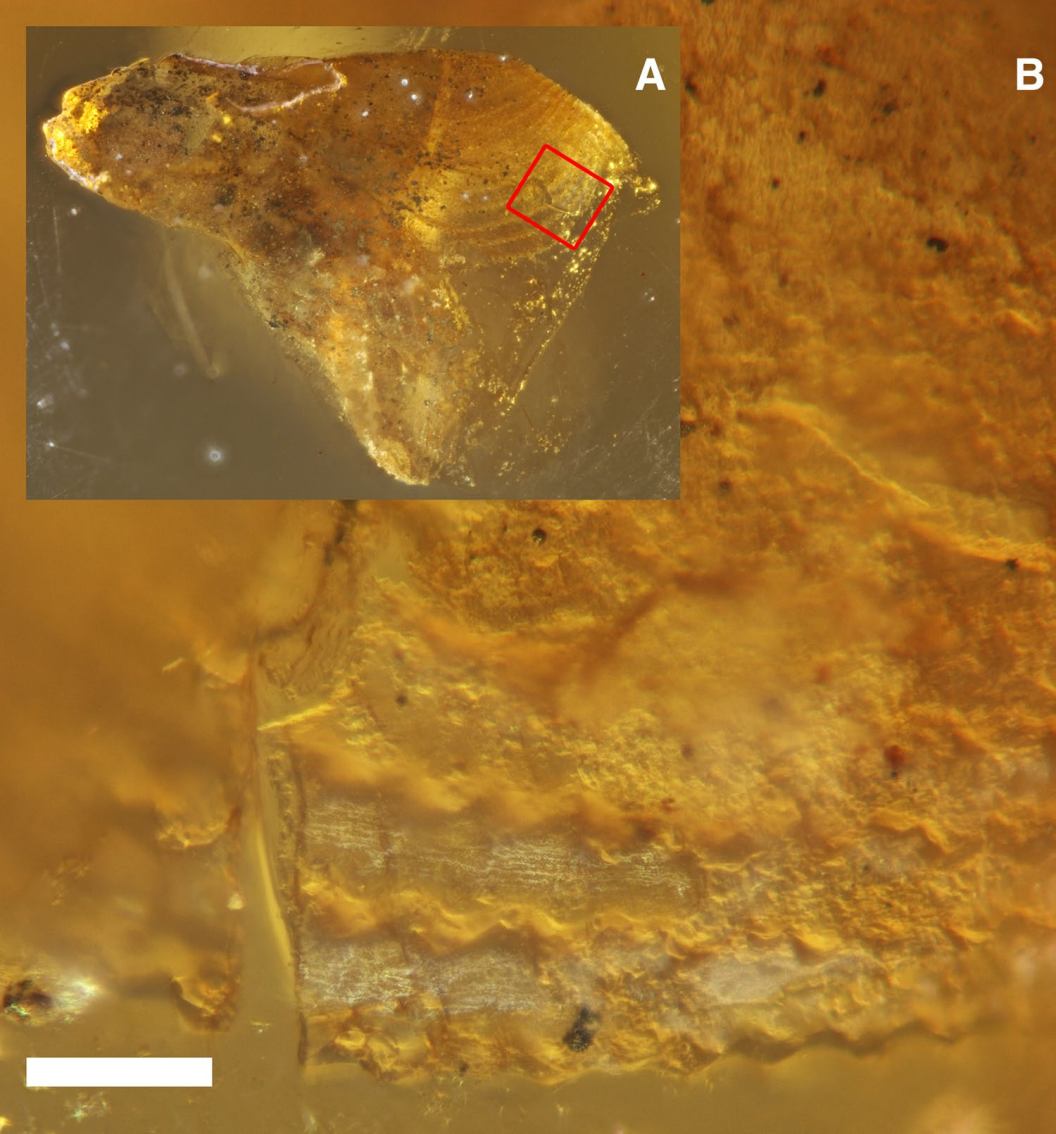
Rasping surface of †*Palaeolignopholas kachinensis* gen. & sp. nov. shell. (**A**) Holotype shell. The red frame marks position of the enlarged area. (**B**) Undulated micro-sculpture of the rasping surface. Scale bar = 100 µm. (Photos: Ilya V. Vikhrev).

**Figure 6:**
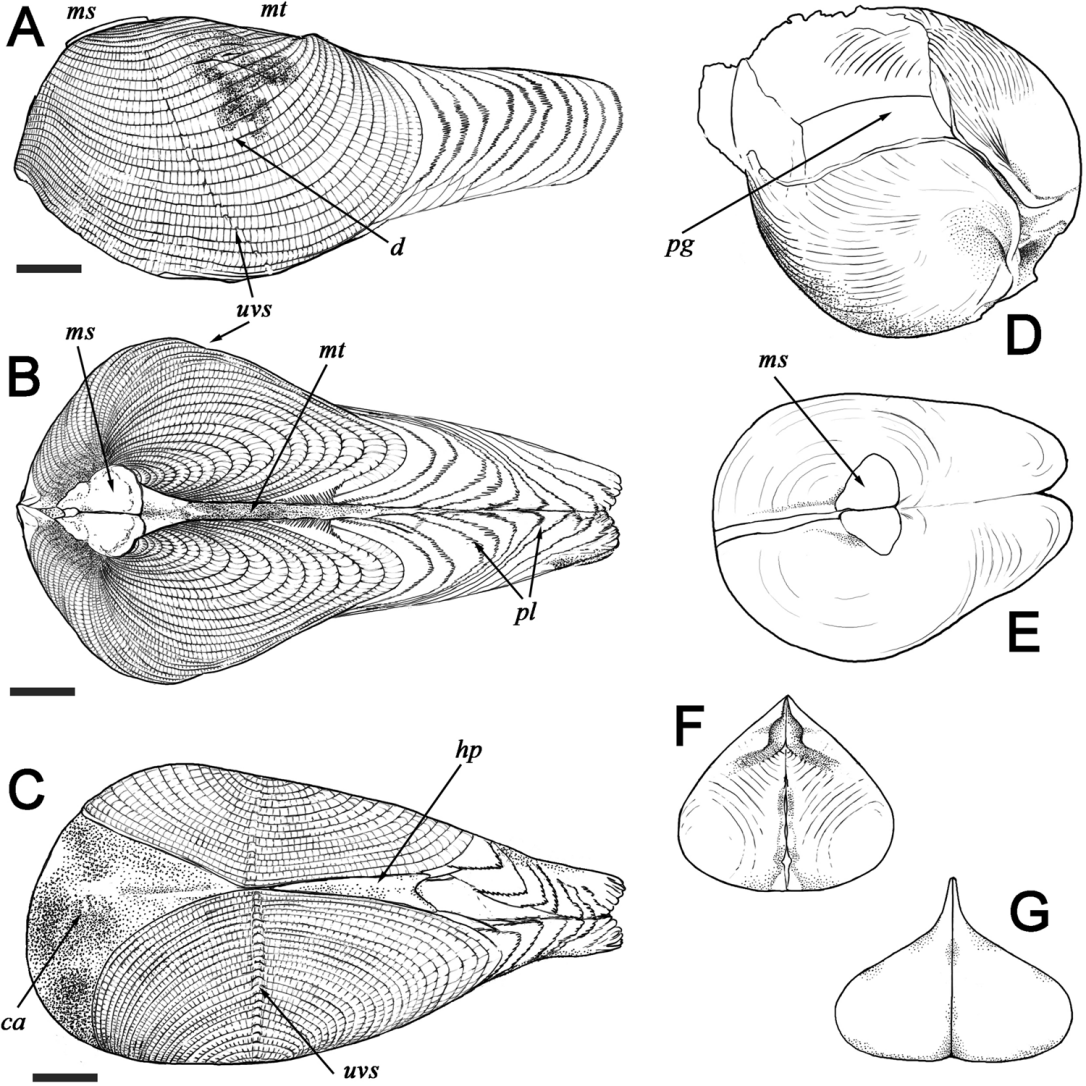
Schematic reconstruction of †*Palaeolignopholas kachinensis* gen. & sp. nov. from lower Cenomanian Kachin amber, northern Myanmar based on the type series and other fossil material^15,16,19,20^. (**A**) Lateral view of adult specimen. (**B**) Dorsal view of adult specimen. (**C**) Ventral view of adult specimen (based on a paratype BMNH 20205^15^). (**D**) Anterio-ventral view of immature specimen. (**E**) Dorsal view of immature specimen. (**F**) Mesoplax of adult specimen. (**G**) Mesoplax of immature specimen. *d* disc; *mt* metaplax; *ms* mesoplax; *hp* hypoplax; *ca* callum; *uvs* umbonal ventral sulcus; *pg* pedal gape; *pl* periostracal lamellae. Scale bars = 1 mm (**A**–**C**). (Line graphics: Yulia E. Chapurina).

**Figure 7:**
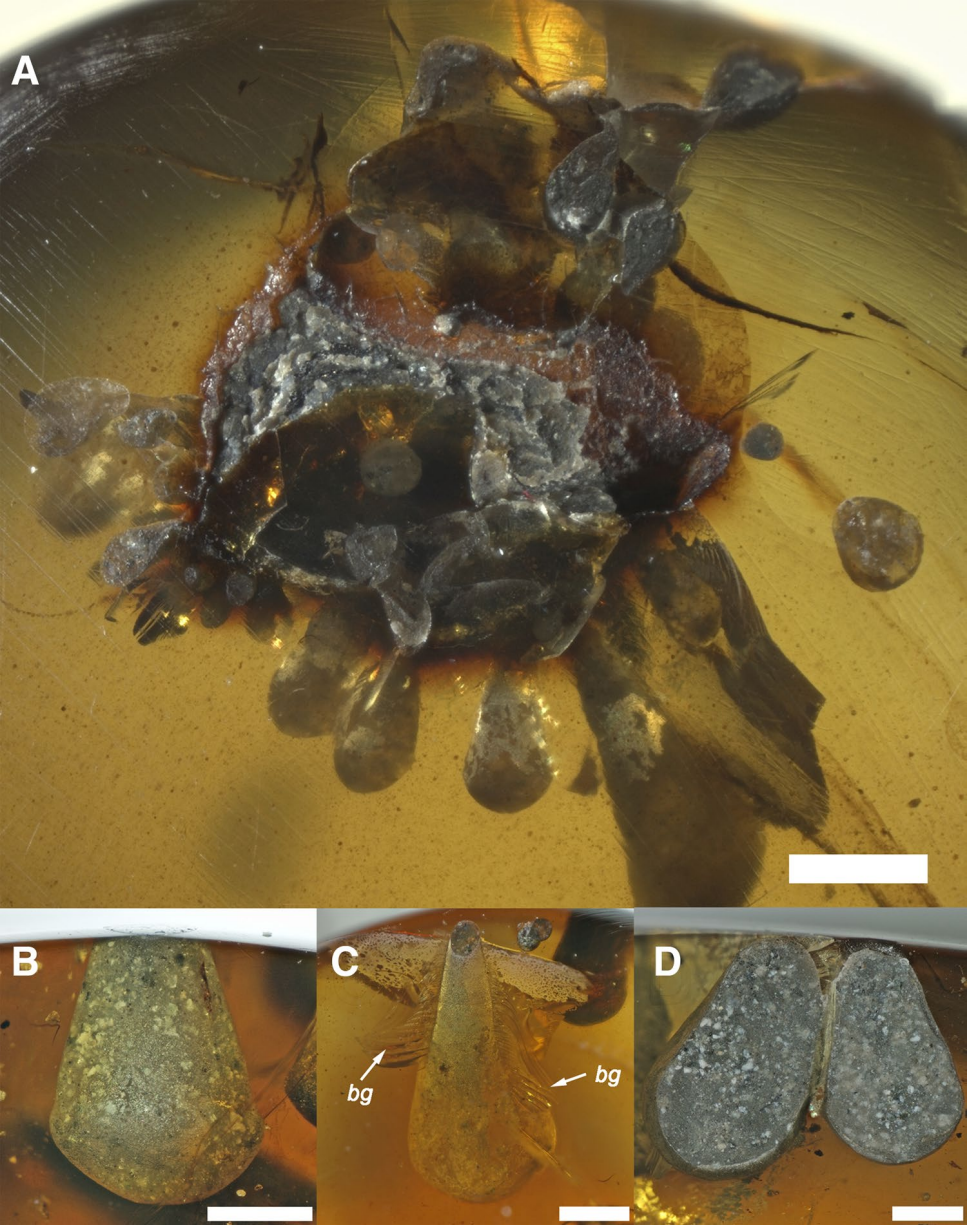
Clavate borings of †*Palaeolignopholas kachinensis* gen. & sp. nov. from lower Cenomanian Kachin amber, northern Myanmar. (**A**) Cluster of borings. It marks drilling of immature piddocks into soft resin from the unidentified plant (wood?) fragment. (**B–D**) Clavate borings of adult piddocks. Scale bars = 1 mm. Abbreviation: *bg* a characteristic bioglyph indicating the shell rotation inside hardening resin. (Photos: Ilya V. Vikhrev).

Figures 1, 2, 3, 4, 5, 6 and 7.

LSID: http://zoobank.org/urn:lsid:zoobank.org:act:F6659EBF-B0A4-4B21-A99B-2C56BDB7EC9B.

Common name. Kachin Amber Piddock.

Holotype. RMBH biv1115, the adult shell with length 3.07 mm and width 1.13 mm “floating” in the resin (Figs. 1A, 3A, 5A,B), local collector leg., Russian Museum of Biodiversity Hotspots, N. Laverov Federal Center for Integrated Arctic Research of the Ural Branch of the Russian Academy of Sciences, Arkhangelsk, Russia.

Paratypes. RMBH biv1116, the fossilized adult shell with length 4.05 mm and width 1.83 mm (Figs. 1C, 3B); RMBH biv1101, the immature specimen with articulated shell (width 1.86 mm) sharing a detached and deflected umbonal paired fragment of the valves due to the shell breakage (Figs. 1B, 4B,C); RMBH biv1101, the other immature specimen with shell length 2.68 mm and shell width 2.52 mm in this amber piece (Figs. 1B, 4A); BMNH 20205, adult specimen [illustrated in Mayoral et al. (2020): fig. 7B^15^], Department of Palaeontology, Natural History Museum, London, UK; NIGP 169623, adult specimen [illustrated in Mao et al. (2018): 100, fig. 8G^20^], and NIGP 169624, two adult specimens [illustrated in Mao et al. (2018): 100, fig. 8H^20^], Nanjing Institute of Geology and Palaeontology, Chinese Academy of Sciences, Nanjing, China; RS.P1450, two sub-adult specimens [illustrated in Smith & Ross (2018): 5, fig. 4A,B^19^], Ru D. A. Smith collection, Kuala Lumpur, Malaysia.

Type locality and strata. The Noije Bum Hill mines, Hukawng Valley, near Tanai (26.3593°N, 96.7200°E), Kachin State, northern Myanmar; Upper Cretaceous (lower Cenomanian; absolute age of youngest zircons in enclosing marine sediment: 98.79 ± 0.62 Ma)^19,22^.

Etymology. The name of this species reflects its type locality, which is situated in the Kachin State of Myanmar.

Diagnosis. As for the genus.

**Figure 8:**
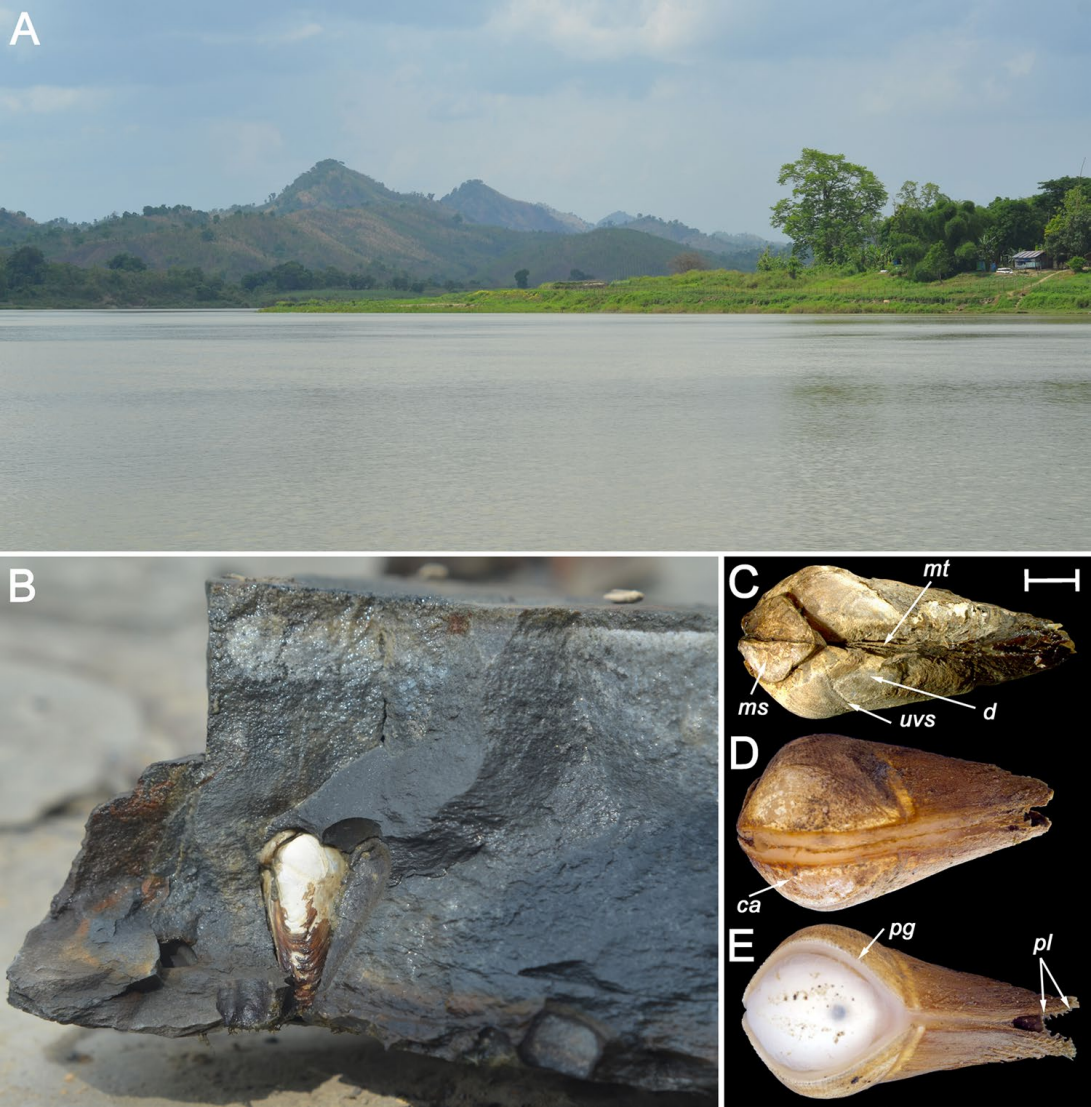
Recent freshwater piddock *Lignopholas fluminalis* (Blanford, 1867) in the middle reaches of the Kaladan River, Rakhine State, Myanmar^13^. (**A**) Habitat of the freshwater piddock: river pool with siltstone rocks at the bottom, a possible modern analogue of the Mesozoic riverine ecosystem with †*Palaeolignopholas*. (**B**) Siltstone rock fragment with living freshwater piddocks inside their clavate borings. (**C**) Ethanol-preserved piddock (dorsal view). (**D**) Living piddock with fully developed callum (ventral view). (**E**) Living piddock with pedal gape (ventral view). Abbreviations: *d* disc; *mt* metaplax; *ms* mesoplax; *ca* callum; *uvs* umbonal ventral sulcus; *pg* pedal gape; *pl* periostracal lamellae. Scale bar = 2 mm. (Photos: Olga V. Aksenova).

Description. Shell small (up to 9.3 mm in length^15,19,20^), conical, with a rounded anterior margin, tapering posteriorly (Figs. 3A,B, 4A–C, 6A–E); its shape is similar to those in the recent *Lignopholas*, *Martesia*, and *Diplothyra*. Valve sculptured, with concave parallel ridges (*Martesia*-like “rasping teeth”) curved anteriorly (Fig. 5A,B). The ridges share a characteristic wave-like micro-sculpture (Fig. 5B). Sulcus deep (Figs. 3A, 4C, 6A–C). Mesoplax longitudinally divided, relatively small, triangular, tapering or lobed anteriorly (Fig. 3A, 6B,F), in immature specimens sometimes with lateral lobes (Figs. 4C, 6E,G). Metaplax and hypoplax long, narrow, not longitudinally divided but sometimes slightly bifurcated posteriorly (Fig. 6A–C). Periostracum densely covered by fine, hair-like lamellae (Figs. 4B,C and 6D). Umbonal reflection with large flattened ridge. Pedal gape presents in immature (Figs. 4A,B, 6D) and some adult specimens (Fig. 3A) but it is covered by callum in older specimens (Figs. 3B, 6C). Morphological details of the new species were also presented in a series of micro-CT images published Mayoral et al. (see Fig. 8 in that paper^15^) and in the reconstruction of Grimaldy et al. (see Fig. 5 in that work^16^).

Borings and corresponding ichnotaxon. The borings produced by †*Palaeolignopholas kachinensis* gen. & sp. nov. represent club-shaped (clavate) structures (Figs. 1C,D, 2A–E, 7A–D), sometimes with a characteristic bioglyph revealing the shell rotation in hardening resin (Fig. 7C). These borings were illustrated in detail^15–17,19,20^, and were considered belonging to *Teredolites clavatus* Leymerie, 1842^15^. Initially, the trace fossils produced by the Kachin amber piddock were described as sporocarps of *Palaeoclavaria burmitis* Poinar & Brown, 2003, a non-gilled hymenomycete taxon^17^. The holotype of this taxon represents a club-shaped piddock crypt labelled as follows: “Amber from the Hukawng Valley in Burma; specimen (in piece B with accession number B-P-1) deposited in the Poinar amber collection maintained at Oregon State University (holotype)^17^”. Hence, *Palaeoclavaria* Poinar & Brown, 2003 and *P. burmitis* Poinar & Brown, 2003 must be considered ichnogenus and ichnospecies, respectively. New ichnotaxonomic synonymies are formally proposed here as follows: *Teredolites* Leymerie, 1842 (= *Palaeoclavaria* Poinar & Brown, 2003 syn. nov.), and *Teredolites clavatus* Leymerie, 1842 (= *Palaeoclavaria burmitis* Poinar & Brown, 2003 syn. nov.).

## Discussion

### Taxonomic placement of the Kachin amber piddock

Previously, †*Palaeolignopholas kachinensis* gen. & sp. nov. was considered a member of the recent genus *Martesia*^15^. However, the latter genus does not share a divided mesoplax and periostracal lamellae^11^, hence, the new species cannot be placed in *Martesia* (nor *Diplothyra*). In its turn, these characters are diagnostic for the genus *Lignopholas*^11–13^. Conversely, *Lignopholas* shares a posterior slope with concentric sculpture, a smooth valve, and a specific shape of periostracal lamellae (being large, flat, and fringed). Therefore, we prefer to place the fossil species from Cretaceous Kachin amber within the new genus †*Palaeolignopholas* that appears to be on the stem lineage of the recent *Lignopholas* clade. The †*Palaeolignopholas* differs from *Lignopholas* by having a posterior slope without concentric sculpture, a sculptured valve with *Martesia*-like “rasping teeth”, and fine, hair-like lamellae.

### Taphonomy of the new fossil taxon

All available specimens and trace fossils of †*Palaeolignopholas kachinensis* gen. & sp. nov. are characterized by very small size^15–17,19,20^. The shell length of the largest specimen ever known is 9.3 mm^20^, while other samples are much smaller^15,19^. For example, the maximum length was 4.2 mm in a comprehensive sample of shells and borings (*N* = 131) examined by Mayoral et al.^15^. At first glance, such a small size might be a diagnostic feature of this taxon, as species from conchologically similar genera such as *Lignopholas*, *Martesia*, and *Diplothyra* share much larger size, usually up to 20–30 mm in length^11,12^. However, *Lignopholas chengi* Turner & Santhakumaran, 1989 was described based on a series of small (possible immature) specimens, the largest of which was 11.5 mm long^11^ that agrees with our dataset on †*Palaeolignopholas*.

It should be noted that fossil specimens of †*Palaeolignopholas kachinensis* gen. & sp. nov. commonly occur “floating” in the amber (see Figs. 1A,B, 3A, 4A–C) indicating that they often bored into freshly produced (liquid or soft) resin^15,19^. Smith & Ross^19^ assumed that these “floating” clams died within 1–2 weeks since their settlement on the resin, while larger examples bored into hardened resin. Mayoral et al.’s taphonomic model^15^ predicts that at first stage the bivalves bored into a living resin-secreting tree, which was periodically immersed in seawater. Later, when the resin hardens, wood can partially decay, being replaced by episodic flows of resin, while the bivalves could repeatedly bore the resin after its hardening^15^. Among recent pholadid taxa, *Martesia nairi* Turner & Santhakumaran, 1989 bores into living mangroves, and damages the trees^11^. However, it was shown that the Kachin amber was produced by Taxodiaceae and Araucariaceae and that several conifer families were the primary sources of Cretaceous amber globally^19,24^. There is not much evidence that conifers such as taxodioids and araucarians could survive in seawater/brackish tidal environments, while the presence of Taxodiaceae pollen in the Noije Bum’s amber horizon^25^ could indicate riparian and freshwater wetland communities^26^. Pyrite inclusions in the Kachin amber pieces^19^ consistent with low oxygenated environments of freshwater and brackish sediments^27^. There are several possible ways of the pyrite origin in freshwater settings, e.g. diagenetic formation from slow oxidation of acid volatile sulphide by organic matter^27^. Hence, we could assume that †*Palaeolignopholas* clams were boring the resin dropped into the water from trunks and branches of conifer trees growing along the downstream river valley, probably having a tidal influence. Our novel records of a bunch of borings situated around a small plant (wood?) inclusion in the amber (see Fig. 7A) and a boring of adult piddock with a specific bioglyph revealing the shell rotation in hardening resin (Fig. 7C) also support this hypothesis.

### Regional paleo-environmental reconstruction

Rare discoveries of supposed marine taxa in the Kachin amber^20,28,29^ were used as a basis to establish the hypothesis on a close proximity of the amber-producing forest to the coastal marine^20^ or brackish water habitats^19^. Records of a juvenile ammonite shell^29^ and several isopods of possible saltwater (littoral or supralittoral) affinities^28,29^ might reveal that the amber was produced from coastal trees located close to a marine bay or other kind of shoreline environments^29^. Numerous records of piddocks and their traces in the amber were also used as a robust evidence supporting the hypothesis on coastal origin of the resin^15,19^. It should be noted that alleged marine gastropods from the Burmese amber^30^ were found to be terrestrial snails from the operculate family Pupinidae (Cyclophoroidea)^31^. A few occurrences of marine oysters, corals, and crinoids adhering to the amber pieces^20^ are not relevant to the paleoenvironmental discussion since they post-date reworking of the amber into a marine environment.

Conversely, the Kachin amber houses a diverse assemblage of freshwater taxa such as caddisflies^32–35^, mayflies^36–39^, stoneflies^40^, chironomids^41^, water measurers^42,43^, odonates^44–48^ and their stem lineage^49^. It is known that insects rarely occur in marine and brackish environments^50^, although recent saltwater-tolerant or even marine species exist among several orders, e.g. Hemiptera^51–53^, Odonata^54–56^, and Trichoptera^57^. Hence, a species-rich and abundant fauna of freshwater insects occurring in the amber could indicate rather a proximity of resin-producing trees to freshwater habitats than to coastal marine environments. Frogs and amphibian egg masses discovered in the Kachin amber^58,59^ are also hardly correspond to saltwater environments.

Based on our new results and the published data, outlined above, we could assume that the Kachin amber-producing forest was partly situated near a downstream (estuarine to freshwater) section of a tropical river. Furthermore, we could hypothesize that these forests as a whole extended across a variety of freshwater and estuarine environments such as coastal rivers, river deltas, lakes, lagoons, and coastal bays. It is known that environmental conditions in downstream sections of rivers draining into the sea are changing periodically from freshwater to slightly brackish or brackish due to the tidal influence^60,61^. There is a possibility of seasonal changes driven by rainfall, when the salinity in lower tidal parts of coastal rivers significantly decreases during the rainy season^62^. Although the Hukawng Valley is currently a mainland site, which is far from the sea, the Burma Terrain (i.e. a microplate covering a large part of Myanmar with amber deposits west of the Sagaing fault) was a tropical island during the Albian–Cenomanian^63,64^. In general, the hypothesis that the resin-producing forest in northern Myanmar was partially situated near a largely freshwater body such as a downstream riverine section could explain the high diversity of freshwater taxa discovered in the Kachin amber. The scarce presence of saltwater (chiefly estuarine) fauna can be explained through occasional marine ingressions into the system. At first glance, habitats of the recent freshwater piddocks *Lignopholas fluminalis* in the middle reaches of the Kaladan River in Myanmar^13,14^ (Fig. 8A–C) can be considered a remote modern analogue of the Mesozoic ecosystem inhabited by †*Palaeolignopholas*. It is known that †*Palaeolignopholas* bored into the amber and wood^15^, while *Lignopholas fluminalis* bores into the bottom rocks^13,14^. The fossil species probably occurred in a downstream (estuarine to freshwater) riverine section, while the recent taxon is known to occur in the middle reaches of a river but may present itself in the estuary as well^13^. Besides, we do not know if they are really related, as the age difference between them is 100 Myr and there is no direct evidence on the missing link available. Theoretically, it could be a homeomorphy. There are other Mesozoic taxa with a portioned mesoplax^21^, providing candidates for an ancestry.

Finally, our novel results not only establish a taxonomic concept for a fossil amber-preserved piddock but also show that the bivalves on their own do not necessarily indicate the presence of brackish to normal marine salinity water.

## Methods

### Data collecting

This study is based on a series of fossil specimens and borings discovered in nine samples of Upper Cretaceous Kachin amber deposited in the Russian Museum of Biodiversity Hotspots, N. Laverov Federal Center for Integrated Arctic Research of the Ural Branch of the Russian Academy of Sciences, Arkhangelsk, Russia. Additional piddock fossils from Natural History Museum, London, UK; Nanjing Institute of Geology and Palaeontology, Chinese Academy of Sciences, Nanjing, China; Ru D. A. Smith collection, Kuala Lumpur, Malaysia; and Division of Invertebrates, American Museum of Natural History, New York, NY, United States of America were examined using photographs presented in published works^15,16,19,20^. The amber samples were collected by local miners in the Hukawng Valley, Kachin State, northern Myanmar. Based on U–Pb dating of zircons from enclosing sediment, the Kachin amber shares the early Cenomanian age^22^.

### Morphological study

Measurements of amber pieces were performed using a digital caliper (Digimatic Coolant Proof, Mitutoyo, Japan). Images of amber pieces were taken using a Canon EOS 6D digital camera (Canon Inc., Japan) with Canon EF 100 mm f/2.8L IS USM macro lens (Canon Inc., Japan). Morphological study of fossil bivalve specimens was based on an approach of Turner & Santhakumaran^11^ with a special focus to the shell shape, size, valve shape and sculpture, the shape of mesoplax, metaplax, hypoplax, and umbonal reflection, and the character of periostracal lamellae. Images and measurements of specimens and trace fossils were taken with a Leica M165C microscope (Leica, Germany).

### Nomenclatural acts

The electronic edition of this article conforms to the requirements of the amended International Code of Zoological Nomenclature (ICZN), and hence the new name and synonymy contained herein are available under that Code from the electronic edition of this article. This published work and the nomenclatural acts it contains have been registered in ZooBank (http://zoobank.org), the online registration system for the ICZN. The LSID for this publication is: urn:lsid:zoobank.org:pub:C14A4E2F-9280-48A6-9C95-8298BD1F964F. The electronic edition of this paper was published in a journal with an ISSN and has been archived and is available from PubMed Central.

## Data Availability

The amber samples containing the type series and borings of the new piddock species are available in the RMBH, Russian Museum of Biodiversity Hotspots, N. Laverov Federal Center for Integrated Arctic Research of the Ural Branch of the Russian Academy of Sciences (Arkhangelsk, Russia); Natural History Museum (London, UK); Nanjing Institute of Geology and Palaeontology, Chinese Academy of Sciences (Nanjing, China); Ru D. A. Smith private collection (Kuala Lumpur, Malaysia), and Division of Invertebrates, American Museum of Natural History (New York, NY, United States of America).
